# Evaluation of the treatment of distal radial volar fracture by different methods sparing the pronator quadratus

**DOI:** 10.1186/s13018-023-04184-8

**Published:** 2023-09-25

**Authors:** Xiaoxia Huang, Boyu Wu, Yimurang Hamiti, Yan Zhao, Yong Teng

**Affiliations:** 1 Graduate School of Xinjiang Medical University, Urumqi, Xinjiang China; 2 Department of Orthopedics, General Hospital of Xinjiang Military Region, Urumqi, Xinjiang China; 3 Department of Microrepair and Reconstruction, The First Affiliated Hospital of Xinjiang Medical University, Urumqi, Xinjiang China

**Keywords:** Distal radius fractures, Volar plating, Pronator quadratus, Sparing, brachioradialis

## Abstract

**Objective:**

The traditional volar approach requires the release of the pronator quadratus (PQ) muscle in the treatment of distal radius fractures. However, intraoperative repair of the PQ muscle often fails due to tissue injury and unstable muscle repair. This study compared the outcomes of different methods of sparing the PQ muscle combined with the volar plate in treating distal radius fractures.

**Methods:**

A total of 95 patients with distal radius fractures sparing the PQ muscle were enrolled with the brachioradialis (BR) splitting approach (group A, 33 people), the volar plating insertion PQ muscle approach (group B, 35 people) and traditional Henry approach without sparing PQ muscle (group C, 27 people). Postoperative internal fixation, fracture healing and postoperative complications were observed in the three groups. The visual analog scale (VAS) of postoperative wrist pain was compared between three groups. The Dienst joint scale was used to evaluate the wrist function of patients, and imaging indexes were used to evaluate the surgical efficacy.

**Results:**

A total of 95 patients with distal radius fractures were followed up for more than one year after surgery. All fractures obtained good union, with no vascular injury, nerve injury or wound infection. Outcomes at three days, one month and three months all showed no significant differences in postoperative imaging indexes among three groups and no significant differences in various indexes among three groups during the same period. The mean operative time in group C was significantly lower than that in groups A and B. There was significant difference in the mean operation time between group A and group B. The amount of mean operative blood loss or mean bone union time in groups A and B was significantly lower than those in group C. No significant difference was shown in mean operative blood loss or mean bone union time between group A and group B. No significant differences in limb function scores, VAS scores and the mean range of motion existed among three groups at the 12-month postoperative follow-up. However, outcomes assessed one week, one month and three months after surgery demonstrated significant differences in the VAS scores and the mean range of motion among three groups, and the group B had lower VAS score and greater the mean range of motion. According to Dienst score, the excellent rate in groups A, B and C was 91.0% (30/33), 94.2% (33/35) and 85.2% (23/27), respectively, at 12 months after surgery. Tendon irritation occurred in 2 cases and joint stiffness in 1 case in group A. In group B, there were 2 cases traumatic arthritis and 2 cases delayed carpal tunnel syndrome and 1 case tendon irritation. In group C, tendon irritation and delayed carpal tunnel syndrome occurred, respectively, in 3 cases.

**Conclusion:**

Our results demonstrated that these two different surgical approaches were effective ways to reserve PQ and had good clinical outcomes. The volar plating insertion PQ muscle approach could reduce early postoperative pain, promote early activity and return to normal life, while the BR splitting approach was more advantageous in intraoperative fracture exposure and could shorten the operative time. However, some defects also existed. At 12 months of follow-up, no significant advantage was seen in sparing the PQ muscle. Therefore, surgeons should be aware of their individual characteristics and choose patients carefully.

## Background

Distal radial fracture is the conjuncture of cancellous and cortical bone, which is a fracture within 3 cm of the distal radius articular surface [1–3]. The incidence of osteoporosis, which easily leads to high loss and fracture fragment comminution after injury, accounts for about 17% of emergency orthopedic patients [4], and the data will improve as our population ages. Injuries involving high levels of energy are common in young patients with sports and traffic-related injuries. As a result of osteoporosis and increased life expectancy, elderly patients frequently sustain low-energy injuries [5]. If left untreated, it will result in a number of complications, such as wrist joint dysfunction, which will negatively impact patients' quality of life. To improve patient's quality of life, there has been an increase in the surgical treatment of distal radius fractures, primarily with volar lock plating fixation. Fixing a fracture and repairing the PQ muscle is controversial [6–9], despite its frequent application in clinical settings. Johnson et al. [10] first reported in 1976 that the PQ muscle stabilizes the radioulnar joint's distal end. Currently, the traditional Henry approach, which requires the surgeon to cut open the pronator muscle, is the method of choice for treating distal radius fractures.

Even in patients with intraoperative repair of the PQ muscle [11], Armangil et al. found a loss of pronation strength after surgery, which may be attributable to tissue injury and edema or inadequate repair. Due to intraoperative amputation of the PQ muscle and the difficulty of suturing, some orthopedic surgeons elect not to repair the PQ muscle following distal radius fracture fixation [12]. In the interim, close suturing of the PQ muscle can result in postoperative ischemic contraction of the wrist, thereby decreasing the wrist's functional range of motion [13, 14]. Therefore, the author considers whether this procedure improves the prognosis of distal radius fractures in the absence of PQ muscle transection.

In 2022, our team reported that the modified Henry approach with sparing pronator quadratus muscle, in which the periosteum was performed with a periosteal stripper to establish a tunnel posterior to the PQ muscle, through which the plate was inserted, can shorten the operation time, intraoperative blood loss and early return to social activities [15]. Kashir et al. [16] believed that the brachioradialis splitting technique for volar plating of distal radius fractures was simple and effective for sparing the PQ muscle. The authors believed that preserving the integrity of the PQ muscle could result in a favorable prognosis.

In this study, 95 patients with distal radius fractures underwent volar plate internal fixation. Intraoperative indexes, postoperative functional efficacy and complications were observed according to different surgical methods. The purpose of this retrospective study was to compare the treatment of three approaches with distal radial volar fracture and compared the clinical, functional and radiological results.

## Methods

Following written approval by an institutional review board, a retrospective clinical study was conducted between October 2020 and March 2022. Ninety-five patients with distal radius fractures who underwent volar plating at the First Affiliated Hospital of Xinjiang Medical University were enrolled in the study. The brachioradialis (BR) splitting approach for volar plating of the distal radius fractures was group A, while group B spared the PQ muscle through the tunnel behind the PQ muscle. The group C was traditional Henry approach without sparing PQ muscle.

The inclusion criteria were as follows: (1) age ≥ 18 years; (2) unstable distal radius fracture due to the presence of dorsal angulation > 20° and shortening > 5 mm or dorsal comminution > 50%; (3) AO classification 23-B, 23-C1 and 23-C2; (4) failure of manual fracture reduction; (5) fresh closed fracture and; (6) with a minimum follow-up of 12 months. The following patients were excluded: (1) ipsilateral or contralateral upper limb fractures and/or dislocation; (2) other diseases of wrist joint function; (3) severe nerve and vascular injury; (4) history of distal radius fracture on the affected side; (5) initial external fixation; (6) mental illness; (7) inability to cooperate on time; (8) AO classification 23-A and 23-C3; (9) failure of manual fracture reduction; (10) time from injury to fracture (> 14 days).

Before the operation based on AO classification, the surgical approach was planned in advance by a professional medical team. The final decision depended on the actual intraoperative situation. If there was no primary injury to the PQ muscle, the fracture was repositioned without dissecting the PQ muscle by the volar plating insertion PQ muscle approach. The same medical team performed all surgical procedures. Three groups had no significant difference in demographics or fracture characteristics (Table 1).

**Table 1:** Baseline characteristics of the three groups

	Group A (33)	Group B (35)	Group C (27)	***P***
Mean age(years)	48.36 ± 13.74	51.97 ± 13.01	55.74 ± 11.90	0.09
Sex				
Male	13	11	9	0.65
Female	20	24	12	
Side of hand				
Left	17	17	12	0.86
Right	16	18	15	
AO classification(n)				
B1	5	9	7	0.85
B2	5	7	7	
B3	8	7	3	
C1	8	7	5	
C2	7	5	5	
Cause of injury(n)				
Fall injury	21	25	16	0.79
Falling injury from height	10	7	9	
Traffic trauma	2	3	2	
Mean interval between injury and operation(days)	3.73 ± 0.80	3.86 ± 1.09	3.78 ± 1.12	0.86

### Surgical procedures

**Fig. 1:**
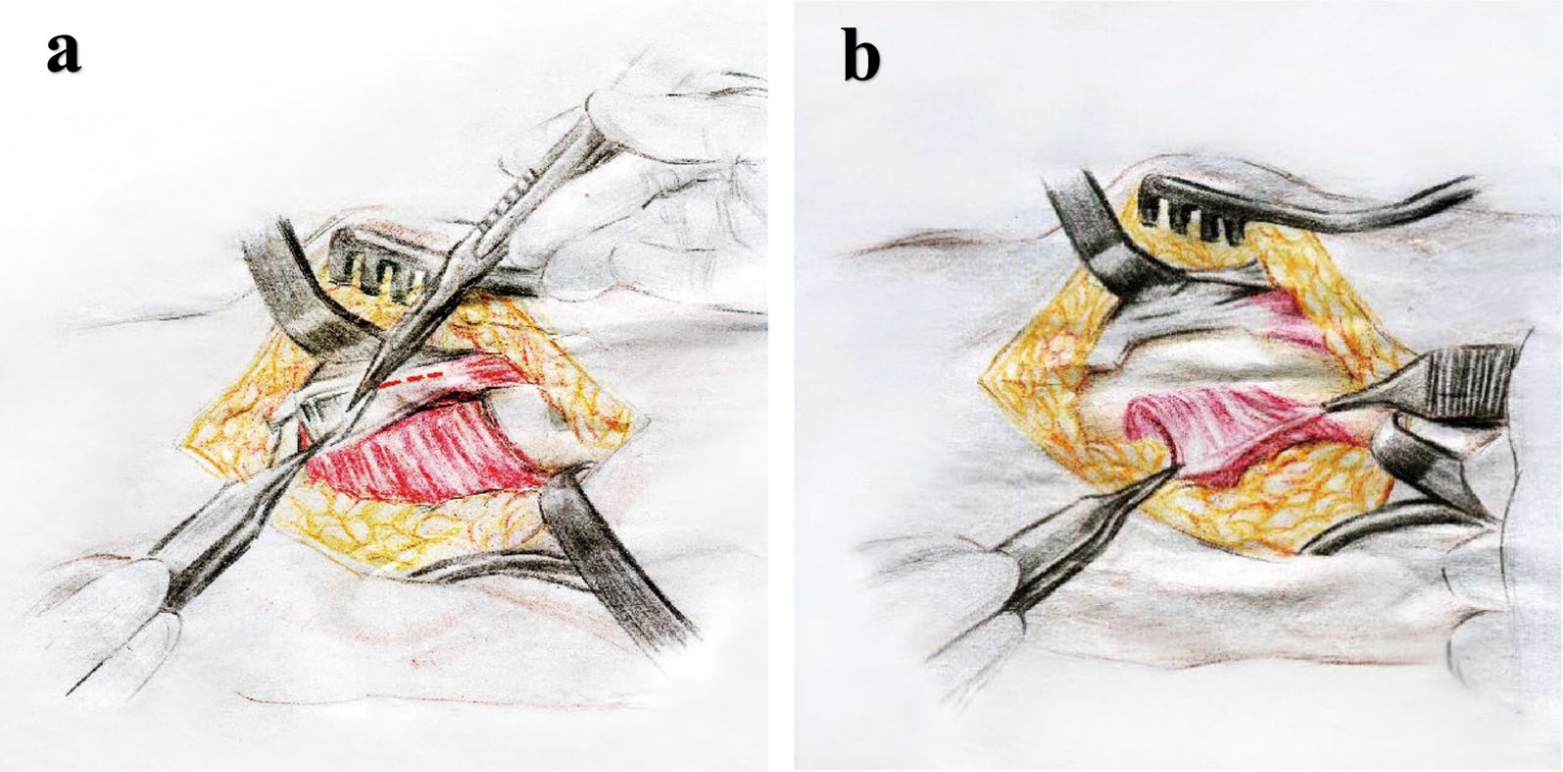
Brachioradialis splitting of the sparing PQ muscle approach. **a** Intraoperative exposure of the PQ/BR complex.** b** The PQ/BR complex was flipped to the ulnar side to fully expose the fracture

**Fig. 2:**
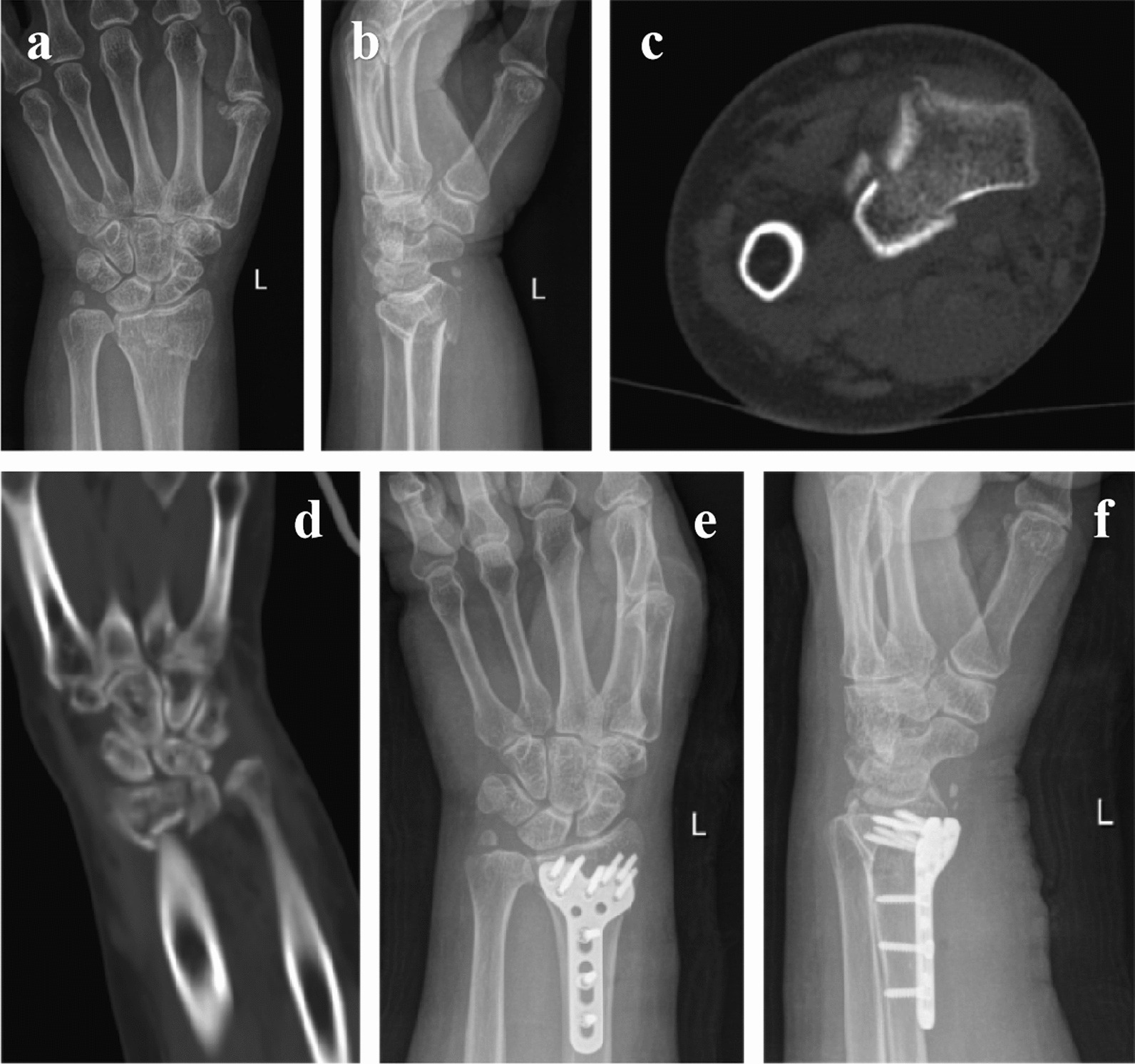
A 55-year-old female patient suffered a distal radius fracture from a fall and underwent the brachioradialis (BR) splitting approach. **a-d** Preoperative imaging of the affected limb. **e**, **f** Postoperative imaging of the affected limb

All patients were placed on the operating table under local anesthesia or general anesthesia and performed by the same team of physicians. For patients in group A, the BR splitting approach through a 5-cm longitudinal incision was made between the flexor carpi radialis and the radial artery. The superficial branch of the radial nerve could be seen below the incision, which should be protected. The radial artery is also identified and pulled to the radial side for protection. The BR tendon was released longitudinally at the attachment of the distal radius (Fig. 1a). The surgeon released the PQ/BR complex together and flipped it to the ulnar side to fully expose the fracture (Fig. 1b). The hematoma and embedded soft tissue of the fracture site were cleared, and 1 ~ 2 Kirschner wires were used to temporarily cross fix the broken end of the fracture. Traction reduction of the fracture was completed under direct vision. X-ray fluoroscopy showed a satisfactory selection of appropriate plate insertion, and the screws were sequentially fixed. Interrupted 3–0 and 4–0 absorbable sutures were used to preserve the PQ muscle by using inter-tendinous anastomosis (Fig. 2).

**Fig. 3:**
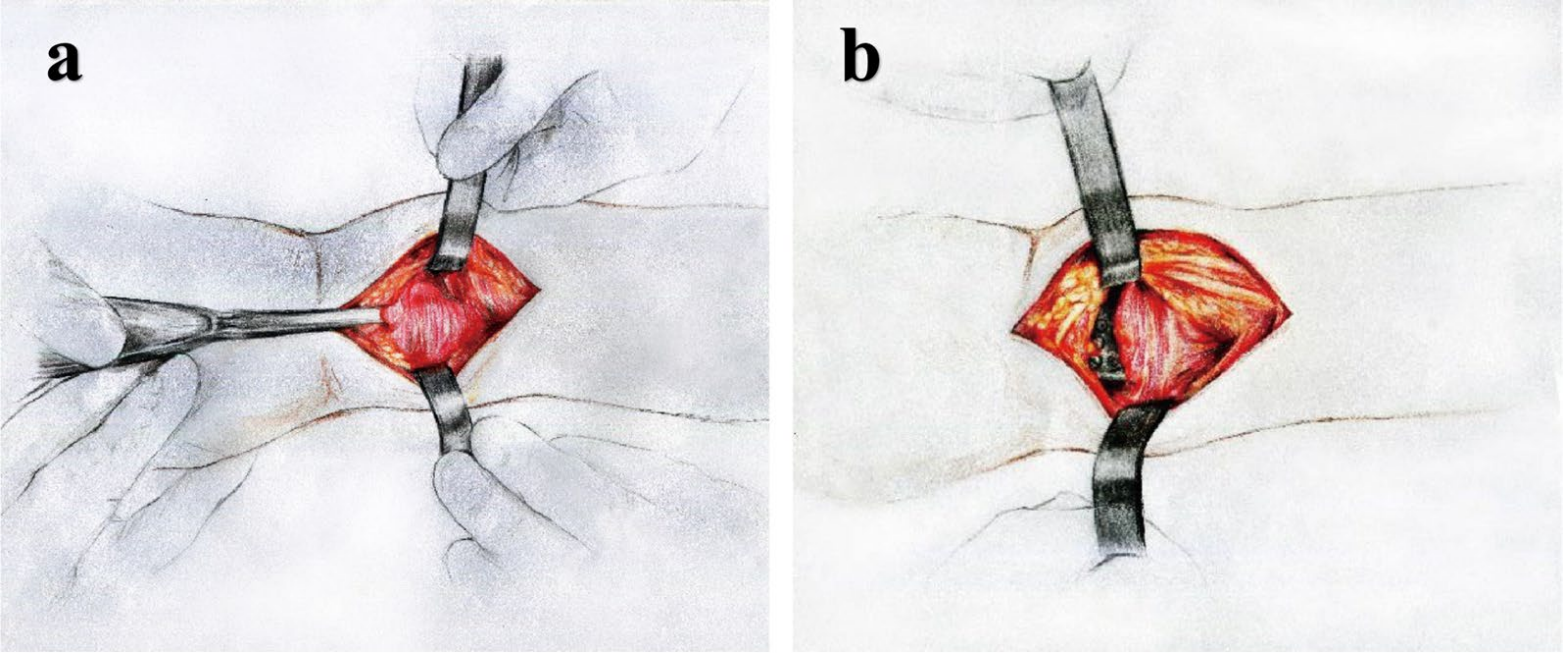
Modified Henry approach. **a** Tunnel was established under the PQ muscle during the operation. **b** The plate was placed below the PQ muscle

**Fig. 4:**
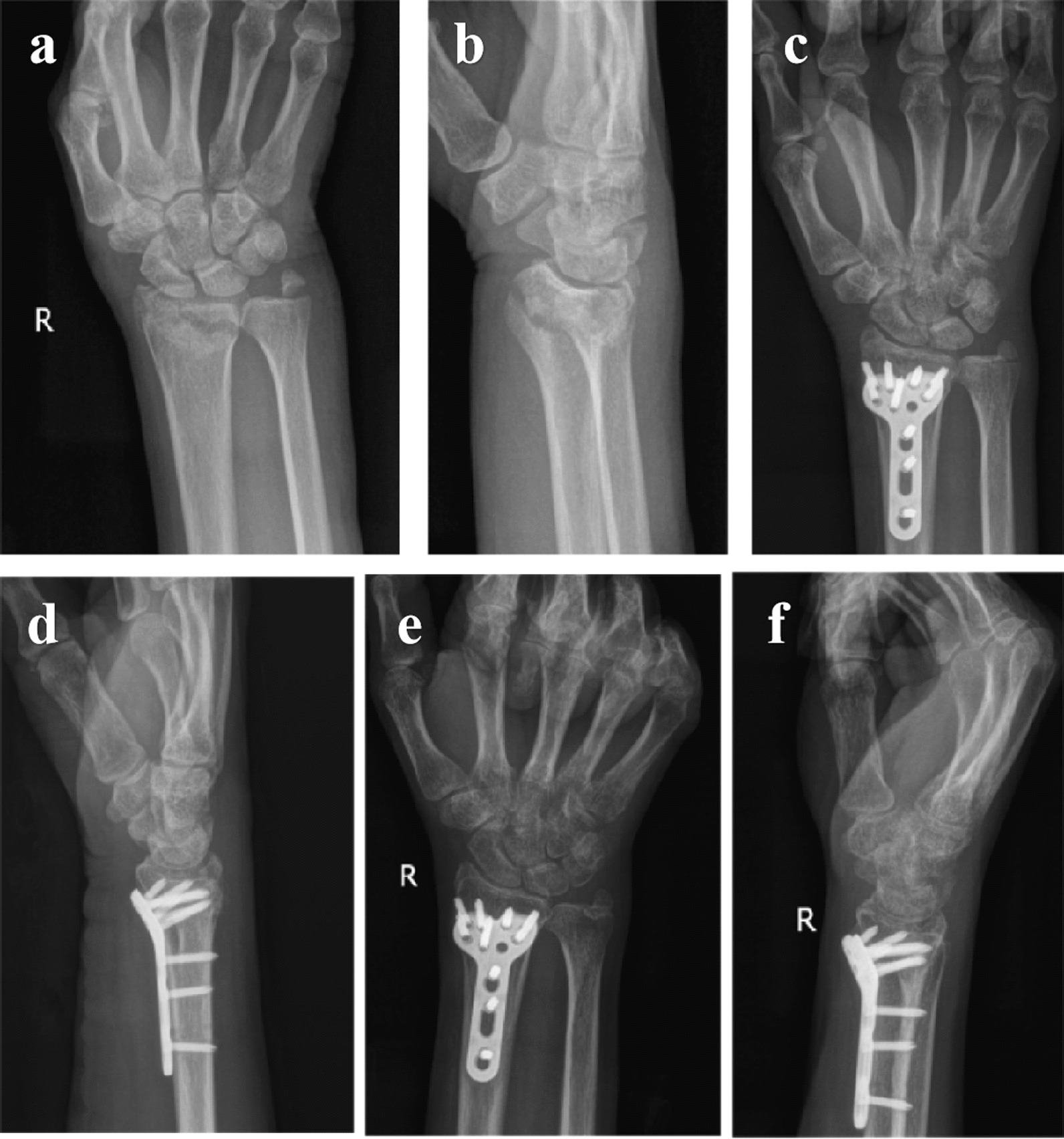
A 56-year-old female patient suffered a distal radius fracture from traffic trauma and performed the modified Henry approach. **a**, **b** Preoperative imaging of the affected limb. **c**, **d** Postoperative imaging of the affected limb in 1 month after surgery. **e**, **f** Postoperative imaging of the affected limb in 3 months after surgery

For patients in group B, the modified Henry approach through the incision between the flexor carpi radialis and brachioradialis was performed to separate the flexor carpi radialis tendon and radial artery vessels. The radial artery was confirmed and retracted radially. The tendon and median nerve were identified and retracted ulnarly. The PQ muscle was utterly exposed. The PQ muscle was then elevated with a periosteal stripper, and a tunnel was established behind the PQ muscle (Fig. 3a). The displaced articular surface and the collapsed dorsal fracture block were reduced, while the wrist joint was subjected to continuous traction with palmar flexion, and the dorsal fracture block was reduced by tendon capsular traction, and excessive thrust pushing was limited. 1 ~ 2 Kirschner wires were used to temporarily cross fix the broken end of the fracture. The articular surface was reconstructed with X-ray fluoroscopy. The distal end of the plate was below the watershed line [17] of the distal radius (Fig. 3b). The PQ muscle was cut lengthwise with a 0.5-cm incision, and the screw was implanted into the anterior rotator muscle corresponding to the hole. The ‘carpal shoot-through view’ can be used to determine whether the screws fixing the metaphysis have penetrated the carpal joint cavity. The tourniquet was released, the operative cavity was rinsed with normal saline, the skin was inserted and drained, the incision was sutured, and a sterile dressing was applied (Fig. 4).

For patients in group C, the steps to expose the PQ muscles are the same as the BR splitting approach. The PQ muscle was exposed and an L-shaped incision was performed along the radial border of the radius to the radial malleolus, and the PQ was then stripped off the radius. After the fragments were repositioned, fluoroscopic confirmation was obtained and a plate was inserted for internal fixation. During the operation, it was found that the PQ muscle had severe edema or damage, which made it difficult to repair, and it was decided to give up the repair.

### Statistical analysis

SPSS Statistics software version 26.0 was applied to the statistical analysis. The mean ± SD was used to represent measurement data. Student's t test was used to compare measurement data (mean operative time, mean operative blood loss, mean bone union time). Single-factor variance test was used to compare the observed values of each index at different groups (mean age, mean interval between injury and operation). The count variables were analyzed by the Chi-square or Fisher's test (sex, side of hand, AO classification, cause of injury, Dienst score, total complication), expressed as a number. Repeated-measures analysis of variance was used to compare the observed values of each index at different time points (radial height, volar tilt, ulnar inclination). *P* < 0.05 was considered statistically significant.

## Result

Ninth-five patients who suffered a distal radius fracture were treated with palmar plating of sparing PQ muscles and enrolled in the study, 33 in group A, 35 in group B and 27 in group C. All fractures were well unioned after up to one year of follow-up.

There was no significant difference in gender, age, side of hand, Ao classification, case of injury and mean interval between injury and operation among three groups (*P* > 0.05) (Table 1).

Evaluation of intraoperative index showed that the mean operative time in group A, group B and group C was 46.82 ± 7.50 min, 54.14 ± 7.22 min, 42.81 ± 4.38 min. It was showed that the mean operative blood loss was 22.72 ± 3.77 ml, 22.00 ± 2.77 ml, 35.18 ± 10.51 ml. The mean bone union time of three groups was 11.42 ± 0.50 weeks, 11.43 ± 0.50 weeks, 11.74 ± 0.52 weeks. The mean operative time in group C was significantly lower than that in groups A and B (*p* < 0.05). There was significant difference in the mean operation time between group A and group B. The amount of mean operative blood loss or mean bone union time in groups A and B was significantly lower than those in group C (*p* < 0.05). No significant difference was shown in mean operative blood loss or mean bone union time between group A and group B (*P* = 0.37 and 0.97, respectively) (Table 2). Outcomes at three days, one month and three months all prompted no significant differences in postoperative imaging indexes (radial height, volar tilt, ulnar inclination) among three groups (*p* > 0.05). There was no significant difference between three groups in the same period (*p* > 0.05)) (Tables 3, 4 and 5).

**Table 2:** Comparison of intraoperative indicators and fracture healing time among patients of each group

	Group A (33)	Group B (35)	*p*	Group A (33)	Group C (27)	*P*	Group B (35)	Group C (27)	*p*
Mean operative time (min)	46.82 ± 7.50	54.14 ± 7.22	0.00	46.82 ± 7.50	42.81 ± 4.38	0.02	54.14 ± 7.22	42.81 ± 4.38	0.00
Mean operative blood loss(ml)	22.72 ± 3.77	22.00 ± 2.77	0.37	22.72 ± 3.77	35.18 ± 10.51	0.00	22.0 ± 2.77	35.18 ± 10.51	0.00
Mean bone union time (weeks)	11.42 ± 0.50	11.43 ± 0.50	0.97	11.42 ± 0.50	11.74 ± 0.52	0.021	11.43 ± 0.50	11.74 ± 0.52	0.021

**Table 3:** Comparison of radius height among patients at various time points postoperatively

	3 days	1 month	3 months	*P*
Group A (33)	11.93 ± 0.58	11.92 ± 0.55	11.88 ± 0.48	0.53
Group B (35)	12.00 ± 0.57	12.00 ± 0.56	11.97 ± 0.51	0.68
Group C (27)	11.91 ± 0.91	12.06 ± 0.81	11.73 ± 0.60	0.32
*P*	0.84	0.06	0.40

**Table 4:** Comparison of palm tilt angles among patients at various time points postoperatively

	3 days	1 month	3 months	*P*
Group A (33)	12.27 ± 0.80	12.24 ± 0.74	12.26 ± 0.77	0.89
Group B (35)	12.27 ± 0.83	12.22 ± 0.77	12.27 ± 0.77	0.91
Group C (27)	12.41 ± 0.88	12.42 ± 0.86	12.48 ± 0.86	0.93
*P*	0.81	0.51	0.94

**Table 5:** Comparison of ulnar deviation in patients at various time points postoperatively

	3 days	1 month	3 months	*P*
Group A (33)	23.28 ± 0.62	23.25 ± 0.61	23.25 ± 0.60	0.95
Group B (35)	23.32 ± 0.57	23.31 ± 0.57	23.30 ± 0.60	0.93
Group C (27)	23.11 ± 0.66	23.04 ± 0.63	23.05 ± 0.62	0.91
*P*	0.67	0.52	0.94	

There was no significant difference in the results of complications among three groups (*P* = 0.13). No intraoperative nerve injury, vascular injury or wound complications were observed in three groups. Tendon irritation occurred in 2 (6.0%) cases and joint stiffness in 1 (3.0%) case in group A. In group B, there were 2 (5.7%) cases traumatic arthritis and 2 (5.7%) cases delayed carpal tunnel syndrome and 1 (2.8%) case tendon irritation. In group C, tendon irritation and delayed carpal tunnel syndrome occurred, respectively, in 3 cases (11.1%) (Table 6). Group B had a slightly excellent rate than group A and C in the Dienst score at the 12-month postoperative follow-up (91.0% vs. 94.2% and 85.2%). However, no significant differences were found among three groups (*P* = 0.56) (Table 6).

**Table 6:** Comparison of Dienst score and complications in patients of each group

	Group A (33)	Group B (35)	Group C (27)	*P*
Dienst score(12 months)				
Excellent	17	23	13	0.56
Good	13	10	10	
Fair	3	2	4	
Excellent rate	91.0%	94.2%	85.2%	
Total complications (n)				
Nerve injury	0	0	0	0.13
Vascular injury	0	0	0	
Wound infection	0	0	0	
Tendon irritation/rupture	2	1	3	
Traumatic arthritis	0	2	0	
Joint stiffness	1	0	0	
Delayed carpal tunnel syndrome	0	2	3	

**Fig. 5:**
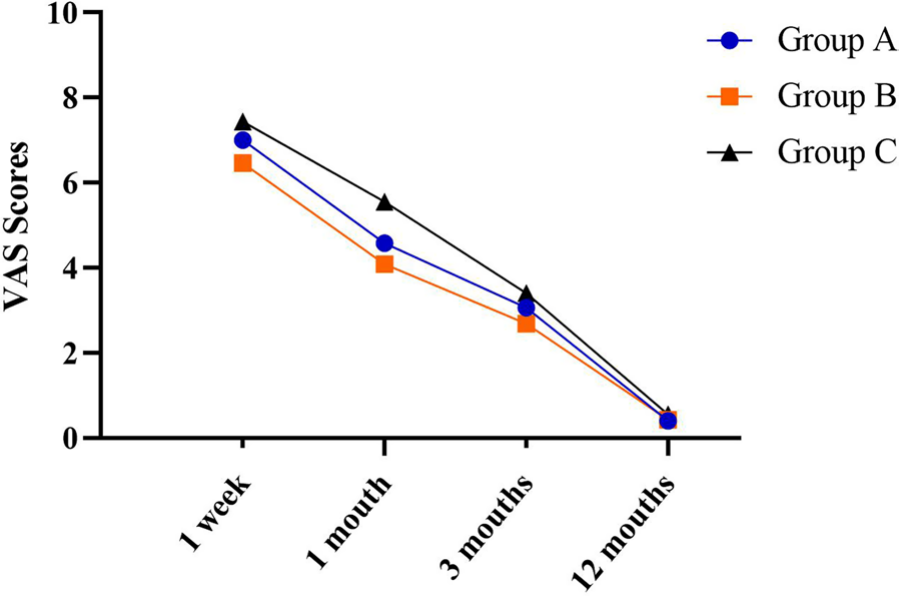
One-year trend in VAS scores for groups A and B

**Fig. 6:**
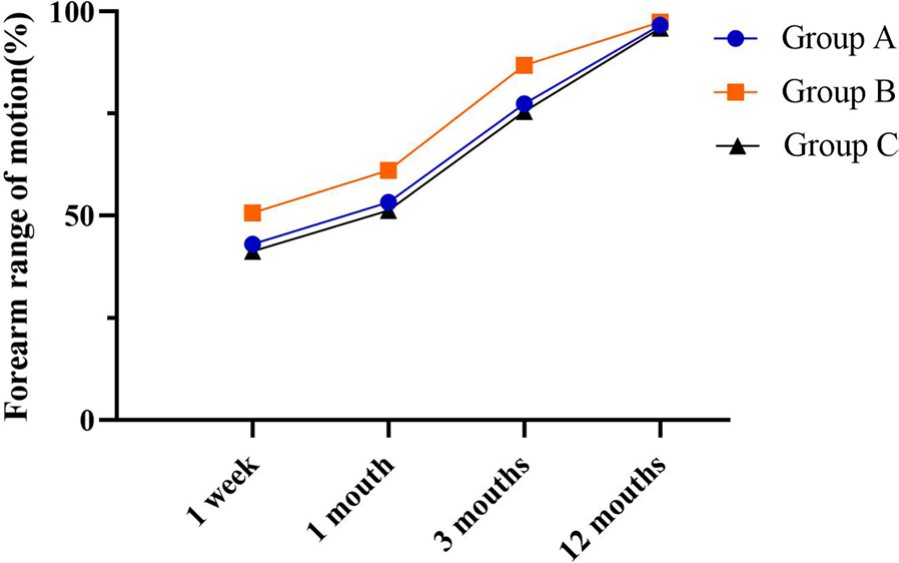
One-year trend in forearm range of motion for groups A and B

The forearm range of motion on affected side in percentage of intact side and VAS scores for each interval is shown in Figs. 5 and 6. The mean values for all variables gradually improved over the year as the range of motion and grip increased and VAS scores decreased. At 12-month follow-up, there were no significant differences in VAS score and forearm range of motion on affected side in percentage of intact side among three groups. However, the evaluation results at 1 week, 1 month and 3 months after surgery showed that there were significant differences in VAS score and forearm range of motion on affected side in percentage of intact side among three groups, among which group B had lower VAS score and forearm range of motion on affected side in percentage of intact side (Tables 7 and 8).

**Table 7:** Comparison of VAS scores among patients at various time points postoperatively

	1 week	1 months	3 months	12 months	*P*
Group A (33)	7.00 ± 0.81	4.58 ± 0.80	3.06 ± 0.56	0.40 ± 0.50	0.00
Group B (35)	6.46 ± 0.66	4.09 ± 0.92	2.69 ± 0.58	0.43 ± 0.50	0.00
*P*	0.00	0.02	0.01	0.76	
Group A (33)	7.00 ± 0.81	4.58 ± 0.80	3.06 ± 0.56	0.40 ± 0.50	
Group C (27)	7.44 ± 0.93	5.55 ± 0.58	3.40 ± 0.57	0.55 ± 0.50	0.00
*P*	0.04	0.00	0.02	0.22	
Group B (35)	6.46 ± 0.66	4.09 ± 0.92	2.69 ± 0.58	0.43 ± 0.50	
Group C (27)	7.44 ± 0.93	5.55 ± 0.58	3.40 ± 0.57	0.55 ± 0.50	
*P*	0.00	0.00	0.00	0.33

**Table 8:** Comparison of forearm range of motion on affected side in percentage of intact side at various time points postoperatively

	1 week	1 months	3 months	12 months	*P*
Group A (33)	43.03 ± 3.29	53.33 ± 3.46	77.42 ± 2.83	96.67 ± 2.98	0.00
Group B (35)	50.71 ± 4.05	61.14 ± 4.04	86.86 ± 3.85	97.43 ± 3.06	0.00
*P*	0.00	0.00	0.00	0.30	
Group A (33)	43.03 ± 3.29	53.33 ± 3.46	77.42 ± 2.83	96.67 ± 2.98	
Group C (27)	41.29 ± 2.23	51.29 ± 3.27	75.55 ± 2.89	95.92 ± 3.11	0.00
*P*	0.02	0.02	0.01	0.35	
Group B (35)	50.71 ± 4.05	61.14 ± 4.04	86.86 ± 3.85	97.43 ± 3.06	
Group C (27)	41.29 ± 2.23	51.29 ± 3.27	75.55 ± 2.89	95.92 ± 3.11	
*P*	0.00	0.00	0.00	0.62	

## Discussion

Previously, non-surgical methods were commonly used for the treatment of fractures. However, there has been a shift in preference toward surgical interventions that aim to restore the anatomical integrity of the fractured area. Various treatment methods, such as percutaneous pinning, external fixation and dorsal and volar plating, have been developed and demonstrated favorable therapeutic outcomes. Plating exhibits several notable advantages over alternative technologies, such as extended exposure of distal radius fractures, precise reduction and stable fixation [15, 18–20]. Dorsal plating was used to buttress many distal radius fractures with dorsal collapse. Its wide application in clinics is limited by attrition from major hardware. Many studies have shown that dorsal plates can lead to the risk of tendon rupture [21, 22]. After 1 year of follow-up examination after surgery, fracture healing usually requires removal of the dorsal plates. Therefore, volar plating is increasingly popular because it can be supported at the distal articular surface to restore the anatomical position. In addition, the system restores and supports the metaphysis, helping to avoid loss of radial slope. At present, AO classification is commonly used worldwide for the preoperative diagnosis of fractures, which is conducive to the design of plate positions in advance. Chauffeur of distal radius pays attention to the injury of scaphoid ligament. The Frykman classification is based on the existence of a fractured ulnar styloid and extension of the fracture to radiocarpal and radioulnar joints. The shortcomings of this classification system are its inability to detect the amount of displacement, fragmentation and resulting shortness. The AO classification of distal radius fractures type A and C3 was excluded from this study. In the case of type A fractures with extraarticular fractures, manual reduction or Kirschner wire fixation is commonly used to protect the blood supply, promote early fracture healing and reduce the financial burden on the patient. For type C3 fractures with complex mass displacement, opening the pronator muscle and performing the surgery under direct vision was recommended to ensure satisfactory reduction and internal fixation. AO classification facilitated preoperative planning and intraoperative evaluation of plate position to avoid tendon irritation in the Soong classification [23, 24]. When fracture fixation conditions permit, positioning with a lower Soong-grade plate minimizes the risk of flexor tendon irritation and rupture. In this study, Soong classification can greatly reduce the interference in the approach through the tunnel behind the PQ muscle.

The PQ muscle is a thick rectangular muscle that arises from the distal ulna and is inserted into the distal radius. The PQ muscle includes superficial and deep components [25]. The superficial head plays a significant role in forearm pronation, and the deep head stabilizes the distal radioulnar joint. The latter prevents triangular fibrocartilage complex (TFCC) damage from impinging the styloid process of the ulna against the carpal bone. Preserving the integrity of the PQ muscle could facilitate blood supply to the area of the fractured distal radius fragment by the anterior interosseous artery [26]. Similarly, this study confirmed that among 95 patients with unstable distal radius fractures, the 27 cases undergoing traditional Henry approach without sparing PQ muscle demonstrated more bleeding, long duration of fracture healing. McConkey et al. [27] demonstrated that the function of the PQ muscle was closely related to pronation torque. The PQ muscles had the advantage of serving as a transfer graft. For example, they could cover the median god meridian during neuroma resection, neurolysis and palmar muscle deficiency. The sparing of the PQ muscle intraoperatively provides sufficient blood supply for fracture healing, confirmed in our previous work [15]. This study compared the outcomes of different methods of sparing the PQ muscle combined with the volar plate in treating distal radius fractures. There was no significant difference in mean bone union time due to the sparing of the anterior pronator muscle by different surgical methods. Nevertheless, there was significant significance in the mean operative time. The BR splitting approach for sparing PQ muscle had advantages over the volar plating insertion PQ muscle approach in exposing the fracture surface. Adequate intraoperative fracture exposure could greatly avoid a series of complications, such as traumatic arthritis, deformities, limited mobility and so on [15, 28].

The traditional Henry approach has been generally accepted by clinicians. The technique is accessed primarily through the interval between the flexor carpi radialis (FCR) and the radial artery. With the Henry approach, the PQ muscle must be released through an L- or Z-shaped incision. The traditional Henry approach without sparing PQ muscle is more dominant in exposing fractures. Therefore, adequate surgical field of view reduces intraoperative fracture reduction time. PQ muscle is a muscle-to-muscle repair, resulting in intraoperative repair difficulties and poor postoperative functional activity. Nevertheless, it has been reported that the repair of PQ muscle is beneficial to the later functional recovery of patients [29]. Whether to repair the pronator muscle intraoperatively and whether the function and efficacy of the postoperative wrist joints are affected are controversial. There were no significant differences in Disabilities of the Arm, Shoulder, and Hand (DASH) and pain scale scores one year postoperatively in a retrospective study [7] and a prospective randomized controlled study [30]. Sonntag [31] demonstrated in a prospective study that PQ muscle repair had no significant advantage in later functional recovery. At the same time, Sandra [9] demonstrated early pain relief with PQ muscle repair in a study of forearm pronation strength in 60 patients. A study on volunteers demonstrated that PQ anesthesia reduced its intensity by 21% [28]. Our previous work has also demonstrated that preserving the pronator muscle allows patients to return to normal life as soon as possible [15]. The anastomosis of the BR splitting approach for sparing PQ muscle through the PQ/BR complex is more robust than that of the traditional Henry approach. The space ulnar pull to the FCR exposed the fracture adequately, avoiding intraoperative damage to the palmar cutaneous branch of the median nerve. The superficial radial nerve and the radial artery were dissected and protected.

Rupture of the flexor tendon of the finger is an uncommon but serious complication after volar plate fixation of distal radius fractures [20]. Schrang et al. [32] suggested that the exposed volar plate was not covered by PQ and the possibility of flexor tendon irritation increased. Flexor tendinitis, including tendon rupture, is a possible complication. Lu et al. [33] advocated that repair of the PQ muscle might have a slight effect on pronation function, but it was not certain whether repair of the pronator muscle was a protective factor to prevent flexor tendon complications. Most clinicians believe that plate placement and PQ muscle maintenance play a significant role in prevention. A cross-sectional study [26] showed that more than 80 percent of surgeons chose to repair the PQ muscle. In addition to maintaining functional and stable components [34], the PQ muscle shield of the plate relative to the flexor tendon is meaningful in preventing tendon irritation or even tendon rupture [35]. Compared to group A and group B, the most common complications for traditional Henry approach without sparing PQ muscle of distal radius fractures include tendon irritation and delayed carpal tunnel syndrome. Symptoms disappeared in three groups after plate removal at the late 1-year follow-up.

This study observed minimal blood loss both prior to skin closure and following tourniquet release, occasionally obviating the necessity for a postoperative drainage system. The observed phenomenon can be attributed to the cushioning effect exerted by the intact PQ muscles on the plate. The PQ muscle assumes a crucial function as a protective barrier, impeding the infiltration of superficial infections into deeper tissues. This idea was confirmed in this study, wound infection did not occur in all patients in this study, and fractures healed well. According to the anatomical measurement of cadaver specimens and CT imaging studies, Jung et al. believed that inserting the distal row screw without cutting into the anterior rotator muscle was sufficient. Compared with other forearm muscles, the length of PQ muscle fibers was shorter, with an average length of 36.6 mm on the surface head and 23.0 mm on the deep head [12, 36]. In addition, overtight repair may result in ischemic contraction of PQ muscles or limit forearm rotation function due to transient dissociation of PQ muscles [37, 38]. Therefore, we believed that performing the surgery without releasing the PQ muscle was beneficial to the rotation function of the patients. The excellent function of the PQ muscle depends on typical anatomical structure. Therefore, the volar plating insertion PQ muscle approach with sparing integrality PQ muscle was superior to other operations in terms of early functional activity.

The objectives of treatment for distal radius fractures include minimizing pain in the wrists and promoting optimal forearm function [39, 40]. With the continuous progress of human society, patients' requirements for quality of life are increasing. In this study, we found a significant difference in average operating time. Compared to group A, the BR splitting approach took less time to treat distal radius fractures. The PQ/BR complex was flipped to the ulnar side, allowing the fracture to be fully exposed to facilitate fracture reduction and fixation. However, the volar plating insertion PQ muscle approach was superior to the BR splitting approach in early pain scores and functional activities. With the exercise of motor function and a decrease in VAS scores, the mean of all the variables improved over the course of the year.

The research possesses notable strengths. This study incorporated a consecutive inclusion of multiple patients classified under the AO system, thereby encompassing a wide range of distal radius fractures [41]. The prevalence of women aged 50 and above suggests a potential bias in the injury mechanism. Furthermore, it is noteworthy that all surgical procedures were executed by an identical medical team. Furthermore, the following data were collected from the study: (1) The BR splitting approach is more complex than the modified Henry approach and carries an increased risk of radial nerve superficial branch and radial artery and brachioradialis tendon; (2) the volar plating insertion PQ muscle approach sparing the PQ muscle can obtain good clinical effects after surgery, requiring detailed anatomical knowledge; (3) the distal end of the plate was below the watershed line of the distal radius; (4) compared with the traditional Henry approach, the incision of the BR splitting approach is 0.5 mm away from the radial side, which is helpful for exposing the brachioradialis tendon; (5) due to individual differences, the brachioradialis tendon in the styloid process of the distal radius is difficult to identify in some patients, but the brachioradialis tendon in the proximal end is easily identified and can be exposed retrogradely to the distal end; (6) implanted screws should be suitable to prevent injury to the dorsal extensor tendon; (7) surgeons can utilize arthroscopy to address concomitant soft tissue injuries [42]; (8) Kirschner wire prying reduction combined with manual assisted reduction; (9) the ‘carpal shoot-through view’ can be used to determine whether the screws fixing the metaphysis have penetrated the carpal joint cavity in the intraoperative fixation of distal radius the fracture; (10) pay attention to how long the tourniquet is in place.

Our study was subject to several limitations. First, the utilization of a retrospective design in this study resulted in the introduction of selection bias. Second, it was a single-center study and only included a subset of patients. We believe larger, multicenter, high-quality, randomized controlled trials could reinforce these conclusions. Third, the AO classification C3 type was not included in this study, which was expected to be overcome in future studies. Finally, we did not follow up with long-term efficacy. In addition, lack of evidence for postoperative verification of the PQ muscles is through magnetic resonance imaging (MRI) in all patients. Finally, the pronator teres and other forearm flexors may affect forearm rotation function and cannot be excluded.

## Conclusion

Our results demonstrated that three different surgical approaches for distal radius fractures provided adequate fixation, satisfactory radiological and functional results for the management. The volar plating insertion PQ muscle approach could reduce early postoperative pain, promote early activity and return to normal life, while the BR splitting approach was more advantageous in intraoperative fracture exposure and could shorten the operative time. However, with the range of motion and grip increased and VAS scores decreased, at 12 months of follow-up, no significant advantage was seen in sparing the PQ muscle. Therefore, surgeons should be aware of their individual characteristics and choose patients carefully.

## Data Availability

The datasets analyzed during the current study are available from the corresponding author on reasonable request.

## Abbreviations

PQ: Pronator quadratus
FCR: The flexor carpi radialis
VAS: The visual analog scale scores
BR: Brachioradialis
TFCC: Triangular fibrocartilage complex
DASH: Disabilities of the Arm, Shoulder, and Hand
MRI: Magnetic resonance imaging
